# Fluctuating asymmetry in brain structure and general intelligence in 73-year-olds

**DOI:** 10.1016/j.intell.2019.101407

**Published:** 2020

**Authors:** Joanna E. Moodie, Stuart J. Ritchie, Simon R. Cox, Mathew A. Harris, Susana Muñoz Maniega, Maria C. Valdés Hernández, Alison Pattie, Janie Corley, Mark E. Bastin, John M. Starr, Joanna M. Wardlaw, Ian J. Deary

**Affiliations:** a School of Psychology and Neuroscience, St Andrews University, St Andrews, UK; b Centre for Cognitive Ageing and Cognitive Epidemiology, The University of Edinburgh, Edinburgh, UK; c Social, Genetic and Developmental Psychiatry Centre, King's College London, London, UK; d Department of Psychology, The University of Edinburgh, Edinburgh, UK; e Centre for Clinical Brain Sciences, The University of Edinburgh, Edinburgh, UK; f Division of Psychiatry, The University of Edinburgh, Edinburgh, UK; g Alzheimer Scotland Dementia Research Centre, The University of Edinburgh, Edinburgh, UK; h Scottish Imaging Network, A Platform for Scientific Excellence (SINAPSE) Collaboration, Edinburgh, UK

**Keywords:** Intelligence, Fluctuating asymmetry, Cortical asymmetry, P-FIT, Fractional anisotropy

## Abstract

Fluctuating body asymmetry is theorized to indicate developmental instability, and to have small positive associations with low socioeconomic status (SES). Previous studies have reported small negative associations between fluctuating body asymmetry and cognitive functioning, but relationships between fluctuating brain asymmetry and cognitive functioning remain unclear. The present study investigated the association between general intelligence (a latent factor derived from a factor analysis on 13 cognitive tests) and the fluctuating asymmetry of four structural measures of brain hemispheric asymmetry: cortical surface area, cortical volume, cortical thickness, and white matter fractional anisotropy. The sample comprised members of the Lothian Birth Cohort 1936 (LBC1936, *N* = 636, mean age = 72.9 years). Two methods were used to calculate structural hemispheric asymmetry: in the first method, regions contributed equally to the overall asymmetry score; in the second method, regions contributed proportionally to their size. When regions contributed equally, cortical thickness asymmetry was negatively associated with general intelligence (*β* = −0.18,*p* < .001). There was no association between cortical thickness asymmetry and childhood SES, suggesting that other mechanisms are involved in the thickness asymmetry-intelligence association. Across all cortical metrics, asymmetry of regions identified by the parieto-frontal integration theory (P-FIT) was not more strongly associated with general intelligence than non-P-FIT asymmetry. When regions contributed proportionally, there were no associations between general intelligence and any of the asymmetry measures. The implications of these findings, and of different methods of calculating structural hemispheric asymmetry, are discussed.

## Highlights

• Previous work has shown links between cortical asymmetry and intelligence.
• In the current study, two methods of calculating global cortical hemispheric asymmetry were compared.
• Equal region contribution: the association between cortical thickness asymmetry and general intelligence was *β* = −0.18.
• Proportional region contribution: no associations between three measures of cortical asymmetry and general intelligence.

## 1 Introduction

Higher general intelligence is associated with educational and occupational successes (Schmidt & Hunter, 1998; Strenze, 2007). Since performance is positively correlated across multiple cognitive tasks, a measure of general intelligence can be estimated using factor analysis (Carrol, 1993; Spearman, 1904). Investigating correlates of general intelligence could provide a better understanding of individual differences in mental ability, and aid identification of people with specific environmental circumstances and disorders that might put them at risk of lower general intelligence. Factors that affect general intelligence have substantial effects during early life (Petrill et al., 2004). For example, shorter gestational time and lower parental socioeconomic status (SES) are reliably associated with lower general intelligence in childhood and adulthood (Davis et al., 2011; Eide, Oyen, Skjaerven, & Bjerkedal, 2007; Hackman & Farah, 2009; Larson, Russ, Nelson, Olson, & Halfon, 2015).

Fluctuating body asymmetry is a measure of developmental instability across species (van Dongen, 2006). As two sides of a bilateral feature (for example the hands or face) represent independent replicates of the same developmental events, random deviations from perfect symmetry in bilateral features indicate minor developmental errors (Hoyme, 1993). Fluctuating asymmetry is thought to be driven by both genetic and non-genetic factors (Leamy & Klingenberg, 2005; Özener, 2010). Fluctuating body asymmetry appears to decrease in early childhood (Hope, Bates, Dykiert, Der, & Deary, 2013) and be negatively associated with childhood SES in older adults (Hope et al., 2013). It is also negatively associated with cognitive performance. For example, children who have more asymmetrical features have slower reaction times (Hope, Bates, Dykiert, Der, & Deary, 2015), and there is an association between neurodevelopmental disorders, such as autistic spectrum disorder and intellectual disabilities, and increased fluctuating body asymmetry (Naugler & Ludman, 1996; Yeo & Gangestad, 2015). Correlation sizes between combined body asymmetry measures (e.g. asymmetries in the widths of wrists, ankles, or elbows, or lengths of fingers) and cognitive performance tend to be small to modest (e.g. Furlow, Armijo-Prewitt, Gangestad, & Thornhill, 1997, *N* = 112, *r* = −0.21; Bates, 2007, *N* = 164, *r* = −0.29). A study with 263 participants found no correlation between fluctuating body asymmetry and cognitive performance (*r* = 0.01; Johnson, Segal, & Bouchard Jr, 2008). However, the age range of was wide (18–79 years-old); relationships between fluctuating asymmetry and general intelligence might be harder to detect amidst the life-long accumulation of structural influences that are not relevant for cognitive outcomes.

Brain asymmetries are not straightforward to interpret, since some functions are specialised in each of the two hemispheres, resulting in some hemispheric asymmetries being positively associated with specific cognitive abilities. For example, Plessen, Hugdahl, Bansal, Hao, and Peterson (2014) report that right > left asymmetry in posterior brain regions is positively associated with visuospatial abilities. Other directional brain asymmetries have negative associations with specific cognitive abilities. For example, greater rightward asymmetry of the fusiform gyrus is associated with increased severity of social cognition deficits in autistic spectrum disorder (*N* = 128, Dougherty, Evans, Katuwal, & Michael, 2016). There is also evidence to suggest that fluctuating (that is, non-directional) brain asymmetry is associated with cognitive performance. Neurodevelopmental disorders such as autistic spectrum disorder, attention deficit disorder, dyslexia and early aggressive behavioural problems are associated with increased fluctuating brain asymmetry (Yeo & Gangestad, 1998). Moreover, Yeo, Ryman, Pommy, Thoma, and Jung (2016), *N* = 244) reported a small negative association (*r* = −0.15) between cortical surface area asymmetry and general intelligence (a latent factor derived from a factor analysis on seven cognitive tests) in young adults.

The parieto-frontal integration theory of intelligence (P-FIT; Jung & Haier, 2007) proposes that cognitive processes rely most heavily on frontoparietal brain regions. In Yeo et al.'s (2016) study, when frontoparietal and non-frontoparietal regions were separated, the association between surface area asymmetry and general intelligence was only found for frontoparietal regions. The authors interpreted this result as being consistent with the P-FIT. However, their study is not decisive, because they did not report whether the association was significantly larger in frontoparietal than non-frontoparietal regions.

The current study aimed to replicate Yeo et al.'s (2016) method with a sample of older adults, while also adding additional brain parameters and providing a methodological alternative for brain asymmetry calculation. Whereas Yeo et al. (2016) focused on the association between brain surface area asymmetry and general intelligence, the current study focused on three measures of brain cortical asymmetry: surface area, volume and thickness. For any associations between brain asymmetry and general intelligence, the role of childhood SES was investigated. Another aim of the current study was to investigate whether cortical fluctuating asymmetry in P-FIT brain regions is more strongly associated with general intelligence than cortical fluctuating asymmetry in non-P-FIT brain regions. Furthermore, the current study compared two methods of calculating cortical fluctuating asymmetry: in the first method, individual regions contribute equally to the overall asymmetry score (used by Yeo et al., 2016); in the second method, the calculation of asymmetry is proportional to the size of the region.

Measurements of brain asymmetry are not limited to grey matter. Some studies have reported associations between specific white matter tract fractional anisotropy directional asymmetries and specific cognitive abilities. For example, Lebel and Beaulieu (2009; *N* = 183) found a significant correlation between leftward lateralization of fractional anisotropy of the arcuate fasciculus and scores on the Peabody Picture Vocabulary Test (PPVT-III; *r* = 0.32; Dunn, 1997). There is a small association between global white matter fractional anisotropy and general intelligence (Penke et al., 2012). However, the association between fluctuating asymmetry in global white matter fractional anisotropy and general intelligence has not been tested before. Therefore, a further aim of the present study was to investigate the relationship between the fluctuating asymmetry of white matter fractional anisotropy across multiple tracts and general intelligence.

## 2 Method

### 2.1 Participants

Participants were members of the Lothian Birth Cohort 1936 (LBC1936, see Deary et al., 2007; Deary, Gow, Pattie, & Starr, 2012; Taylor, Pattie, & Deary, 2018). The current study uses Wave 2 of data collection (collected between 2007 and 2011, Age *M* = 72.9 years, *SD* = 0.71), which was the first wave at which brain MRI scans were collected. Of those participants who completed cognitive testing at recruitment (Wave 1; *N* = 1091), 731 participants agreed to brain scanning at Wave 2. All participants were scanned in the same scanner in the same clinic.

After image processing, MRI data from 636 participants (336 males, 300 females, Age: *M* = 72.7 years, *SD* = 0.73) were available, and are the subject of this report. Depending on cognitive test, data from *N* = 624–636 was available (see Table 1). For the white matter fractional anisotropy analysis, after diffusion MRI processing, data from 556 to 664 participants were available depending on the tract of interest (see Supplementary Table 1).

**Table 1:** Descriptive statistics for cognitive tests (all completed at age 73).

Cognitive domain	Test	*N*	*M* (*SD*)
Visuospatial skills	Matrix Reasoning	634	13.52 (4.93)
	Block Design	634	34.38 (10.01)
	Spatial Span	634	14.79 (2.72)
Crystallised ability	NART	634	34.66 (8.10)
	WTAR	634	41.27 (6.94)
	Phonemic Verbal Fluency	635	43.55 (12.78)
Verbal memory	Verbal Paired Associates	623	27.57 (9.48)
	Logical Memory	635	75.03 (17.84)
	Digit span backwards	636	7.88 (2.31)
Processing speed	Symbol Search	634	24.88 (6.05)
	Digit-Symbol Substitution	634	56.68 (11.79)
	Inspection Time	624	111.78 (10.95)
	Four-Choice Reaction Time (s)	635	0.64 (0.08)

An additional analysis was also run that excluded participants who had strokes or visible abnormalities in MRI images (e.g. cists); for this, the *N* = 530. The result of this analysis was very similar to that of the full analysis, and is presented in Supplementary Table 2.

Ethical permission for the LBC1936 study was obtained from the Multi-Centre Research Ethics Committee for Scotland (MREC/01/0/56), the Lothian Research Ethics Committee (LREC/2003/2/29) and the Scotland A Research Ethics Committee (07/MRE00/58). All participants gave written consent before cognitive and MRI measurements were collected.

### 2.2 Measures

#### 2.2.1 Cognitive tests

The participants completed a wide-ranging selection of cognitive tests, of which 13 were selected for use in the current study. All tests were individually administered and all participants were tested in the same location, using the same equipment and instructions. Based on previous analyses of this battery of cognitive tests (e.g. Ritchie et al., 2016), these tests were grouped into four cognitive domains, modelled in a confirmatory factor analysis-based hierarchical model with a second-order general factor (general intelligence): Visuospatial Skills, Crystallised Ability, Verbal Memory and Processing Speed.

*Visuospatial Skills* consisted of two subtests from the Wechsler Adult Intelligence Scale-III (WAIS-III; Wechsler, 1997a): Matrix Reasoning and Block Design. It also included the Spatial Span (average of forward and backward) subtest from the Wechsler Memory Scale-III (WMS-III; Wechsler, 1997b).

*Crystallised Ability* was measured by two tests involving the participant reading a list of irregular words out loud: the National Adult Reading Test (NART; Nelson & Wilson, 1991) and the Wechsler Test of Adult Reading (WTAR; Wechsler, 2001). A test of Phonemic Verbal Fluency (Lezak, Howieson, Loring, Hannay, & Fischer, 2004) was also included.

*Verbal Memory* was measured using two subtests from the WMS-III: Verbal Paired Associates (total from immediate and delayed tests) and Logical Memory (total from immediate and delayed tests). It also included the Digit Span Backwards subtest from the WAIS-III.

*Processing Speed* was measured by two pencil and paper tests from the WAIS-III: Symbol Search and Digit-Symbol Substitution. Furthermore, two computerised instruments were used: Inspection Time (Deary et al., 2004); and Four-Choice Reaction Time (Deary, Der, & Ford, 2001).

#### 2.2.2 Childhood SES measures

The childhood SES data were collected when participants were recruited as members of the LBC1936, at a mean age of 70 years. The four measures relate to when participants were about 11 years old. These measures are: number of people per room in their house; type of toilet (indoor or outdoor) which is indicative of the size and quality of a house in the 1930s (indoor toilet was scored as higher SES; Dedman, Gunnell, Davey Smith, & Frankel, 2001); number of people sharing a toilet; and father's social class. Father's social class was measured using the UK's 1951 Classification of Occupations (General Register Office, 1956; Knight, 1967). This was compiled for use in connection with the 1951 Census of England, Wales and Scotland and generally coincided with the middle of the father's career. It is reported on a 5-point scale ranging from 1 = professional to 5 = unskilled.

#### 2.2.3 MRI protocol

For full details of the MRI protocol, see Wardlaw et al. (2011). In brief, MRI data was collected in the Brain Research Imaging Centre, University of Edinburgh, using a GE Signa LX 1.5T clinical scanner (General Electric, Milwaukee, WI). Image acquisition comprised whole brain T2-weighted, T2*-weighted and FLAIR-weighted axial scans, and a high-resolution T1-weighted volume sequence in the coronal plane. Single-shot, spin-echo, echo-planar, and diffusion-weighted volumes (b = 1000 s/mm^2^) were acquired in 64 non-collinear directions along with seven T2-weighted volumes (b = 0 s/mm^2^). Seventy-two adjacent 2 mm thick axial slices acquired with a field of view of 256 × 256 mm and a matrix size of 128 × 128, giving a resolution of 2 × 2 × 2 mm^3^. Repetition and echo times were 16.5 s and 95.5 milliseconds, respectively. Total image acquisition took approximately 70 min.

Methods for cortical reconstruction and volumetric segmentation were performed with the FreeSurfer image analysis suite (http://surfer.nmr.mgh.harvard.edu/). This FreeSurfer parcellation yields 34 paired measures across the two hemispheres based on the Desikan-Killiany atlas (Desikan et al., 2006). It was used to acquire a left and right measure for 34 regions for surface area, volume and thickness (for descriptive statistics, see Supplementary Table 3). The resultant parcellations were then visually inspected and manual editing rectified issues with skull stripping, tissue identification or ROI boundary identification. Thirty-two participants were excluded at this stage due to infarct, poor quality scan, general brain tissue identification failure, or major parcellation failure.

### 2.3 Tractography protocol

**Fig. 1:**
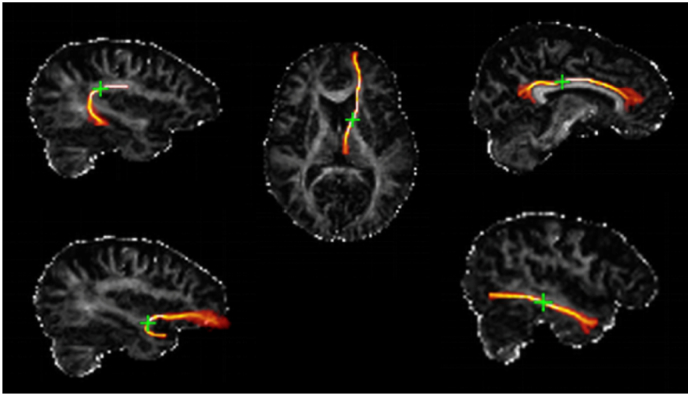
White matter tracts, segmented using probabilistic neighbourhood tractography overlaid on fractional anisotropy maps for a representative participant. Tracts are shown in orange and seed points are indicated by a green cross. Top (left to right): arcuate, anterior thalamic radiations, bilateral cingulum cingulate gyri. Bottom (left to right): uncinate, inferior longitudinal fasciculi (adapted from Ritchie et al., 2015). (For interpretation of the references to colour in this figure legend, the reader is referred to the web version of this article.)

In the current study, bilateral anterior thalamic radiations, cingulum bundles, and arcuate, uncinate, and inferior longitudinal fasciculi were used (Fig. 1); the splenium and genu of the corpus callosum were also identified from this protocol but were not used in the current analysis as they are not separable for left and right hemispheres.

For full details of the tractography protocol, see Clayden et al. (2011) and Bastin et al. (2010). In brief, data were pre-processed to extract the brain, remove bulk participant motion and eddy current-induced distortions, and estimate water diffusion tensor parameters using FLS tools (FMRIB, Oxford UK; Smith et al., 2004). Brain connectivity data were created using the BedpostX/ProbTrackX tractography algorithm (Behrens, Berg, Jbabdi, Rushworth, & Woolrich, 2007) with its default parameters of a 2-fiber model and 5000 streamlines to reconstruct tracts of interest. For each participant, the seed point producing the best match tract to a reference for each of the 10 pathways was determined using probabilistic neighbourhood tractography, implemented in the TractoR package (Clayden et al., 2011), with the resulting tractography mask applied to each participant's mean diffusivity and fractional anisotropy volumes. Tract-averaged values (weighted by the connection probability) were calculated from these masks and used in all subsequent analyses. The image analysts were blind to the characteristics of each participant.

### 2.4 Statistical analysis

All analyses were conducted in R (version 3.2.5; R Core Team, 2016). The lavaan package (Rosseel, 2012) was used to estimate structural equation models. The following fit indices were considered: chi-squared (χ^2^), Comparative Fit Index (CFI), Tucker Lewis Index (TLI), Root Mean Square Error of Approximation (RMSEA) and Root Mean Square Residual (SRMR). Hu and Bentler's (1999) criteria for acceptable model fit were as follows: CFI > 0.95, TLI > 0.95, RMSEA < 0.06, SRMR < 0.08. Using these criteria, all models were estimated using full information maximum likelihood. We also tested the main associations of interest using a Bayesian analysis. To do so, we extracted the factor scores for general intelligence from the structural equation model and ran a Bayesian correlation to link them with each brain asymmetry variable. We did so using the BayesFactor package for R (Morey & Rouder, 2018). In this way, we were able to calculate the strength of the evidence for and against the null hypothesis (the latter being that there was no association between the brain asymmetry variable in question and general intelligence).

### 2.5 Calculation of asymmetry

Two methods were used for calculating fluctuating asymmetry. The first was that described by Yeo et al. (2016, p. 95). This method aimed to calculate fluctuating asymmetry for each of the four measures: cortical surface area, cortical volume, cortical thickness and white matter fractional anisotropy parameters with each region/tract contributing equally to the overall asymmetry score. Yeo et al. (2016) explain that this modelled measure of equal contribution is typical for aggregate measures based on body features (e.g. Bates, 2007; Furlow et al., 1997). The procedure involved the following steps:

1. For each participant, directional asymmetries were calculated for each region/tract (the right value was subtracted from the left; see Supplementary Fig. 1);
2. The mean directional asymmetry for each region/tract was found across the whole sample;
3. The values in Step 2 were subtracted from the values in Step 1, providing a measure of deviance from the sample mean for each region/tract for each participant;
4. The absolute values of the values in Step 3 were taken, providing a non-directional measure of asymmetry;
5. These values were divided by the average of each participant's left and right hemisphere values for the relevant region/tract, ensuring that each region/tract contributed equally to the overall asymmetry score.

• This method treats each parcellation of the brain as an equal unit of interest.
• This step was used by Yeo et al. (2016) since regions vary in size (e.g. in this sample, the total surface area of the superior frontal region is 12,730.15 mm^2^ whereas the total surface area of the entorhinal region is 722.90 mm^2^). Absolute asymmetry scores for each cortical region are given in Supplementary Table 4.

6. The values for all regions/tracts were averaged for each participant, providing an overall asymmetry score for each participant;
7. In separate analyses, testing the P-FIT theory (Jung & Haier, 2007), separate asymmetry scores (using the procedure above) were found for each participant for P-FIT and non-P-FIT regions (see Supplementary Figs. 2 and 3).

• P-FIT regions: caudal middle frontal, frontal pole, fusiform, inferior parietal, lateral orbitofrontal, medial orbitofrontal, rostral middle frontal, superior frontal, superior parietal and supramarginal.
• Non-P-FIT regions: bank superior temporal sulcus, caudal anterior cingulate, cuneus, entorhinal, inferior temporal, insula, isthmus cingulate, lateral occipital, lingual, middle temporal, parahippocampal, paracentral, pars opercularis, pars orbitalis, pars triangularis, pericalcarine, postcentral, posterior cingulate, precentral, precuneus, rostral anterior cingulate, superior temporal, temporal pole and transverse temporal.

Although it might be valid for body-part asymmetry scores to make equal contributions to overall asymmetry scores, the same might not be the case for the brain. It might not be appropriate to allow, for example, the entorhinal cortex (722.90 mm^2^) to contribute as much to the overall asymmetry measure as the much larger superior frontal region (12,730.15 mm^2^). Allowing an equal contribution of regions could result in a substantially larger asymmetry score than is representative of the entire cortex. It is possible that proportional asymmetry provides a more representative index of hemispheric asymmetry. Thus, our second method for calculating fluctuating asymmetry scores involved each region contributing proportionally to the asymmetry score, depending on their size. For this method, for each measure (cortical surface area, volume and thickness), the total right hemisphere value was subtracted from the left.

## 3 Results

All models reported in this section had acceptable fit, according to the criteria in the Methods section (see Supplementary Table 5).

### 3.1 Cognitive descriptive statistics

Descriptive statistics for all 13 cognitive tests are presented in Table 1 for the 636 participants who completed cognitive tests and MRI scanning.

Tests for measurement invariance were performed (see Widaman, Ferrer, & Conger, 2010). For general intelligence, strong measurement invariance for males and females could not be assumed (*p* < .001 for the difference between the model with strong invariance and one with only configural invariance). Therefore, the latent factor of general intelligence could not be treated the same across the sexes (see Supplementary Tables 6 and 7). Consequently, sex differences were not investigated in the models that included the latent factor of general intelligence.

### 3.2 Model of general intelligence

A hierarchical confirmatory factor analysis model was estimated for general intelligence (see Supplementary Fig. 4). Each test loaded highly on the relevant domain, and all domains had high loadings on general intelligence (see Fig. 3). In this model, the residual variance of the path from general intelligence to Verbal Memory was near-zero and was estimated as negative (*β* = −1.41), indicating that all variance in Verbal Memory was shared with general intelligence. To allow the model to converge on within-bounds estimates, the variance of Verbal Memory was fixed at zero. Covariance paths were added between NART and WTAR and between Verbal Paired Associates and Logical Memory, as these tests are similar and share method variance not incorporated by the rest of the model. All paths were statistically significant at the *p* < .001 level (see Supplementary Table 8).

### 3.3 Regional cortical asymmetry and associations with intelligence

**Fig. 2:**
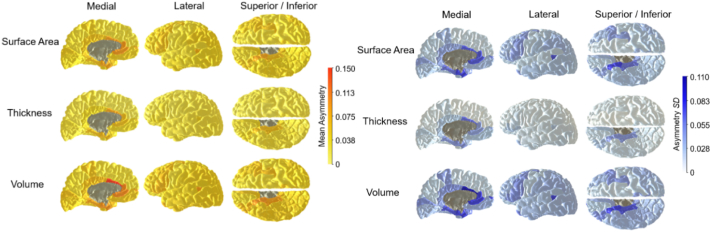
Brain heatmaps illustrating the absolute asymmetry of the 34 cortical regions: Means (left) and standard deviations (right).

Descriptive statistics for the left and right hemisphere surface area, volume, and thickness are presented in Supplementary Table 3. First, simple directional asymmetries (left minus right) were computed for each cortical region for each participant (see Supplementary Fig. 1). To evaluate the extent and significance of these directional asymmetries, one-sample *t*-tests were conducted, comparing each asymmetry value to zero. Then, the absolute asymmetries of the 34 cortical regions were calculated (see Fig. 2 and Supplementary Table 4).

*β*-weights of paths from the absolute asymmetry of the 34 cortical regions to general intelligence are presented in Supplementary Table 9. To summarise, for surface area asymmetry, the precuneus (*β* = 0.13, *p* = .007), rostral anterior cingulate (*β* = 0.13, *p* = .004) and transverse temporal (*β* = 0.09, *p* = .047) regions were positively associated with general intelligence. For cortical volume, asymmetry in the inferior temporal region was positively associated with general intelligence (*β* = 0.10, *p* = .034). For thickness asymmetry, there were no significant associations between any of the 34 cortical regions and general intelligence.

#### 3.3.1 Equal regional contribution analysis: general intelligence model

An equal regional contribution analysis was conducted, as in Yeo et al. (2016). Collapsing across the 34 cortical regions, values of overall asymmetry were calculated for surface area, volume and thickness. Surface area asymmetry was strongly correlated with volume asymmetry (*r* = 0.72, *p* < .001), and volume asymmetry was modestly correlated with thickness asymmetry (*r* = 0.28, *p* < .001). But, as was also found by Koelkebeck et al. (2014), there was no significant correlation between surface area asymmetry and thickness asymmetry (*r* = 0.04, *p* = .377).

**Fig. 3:**
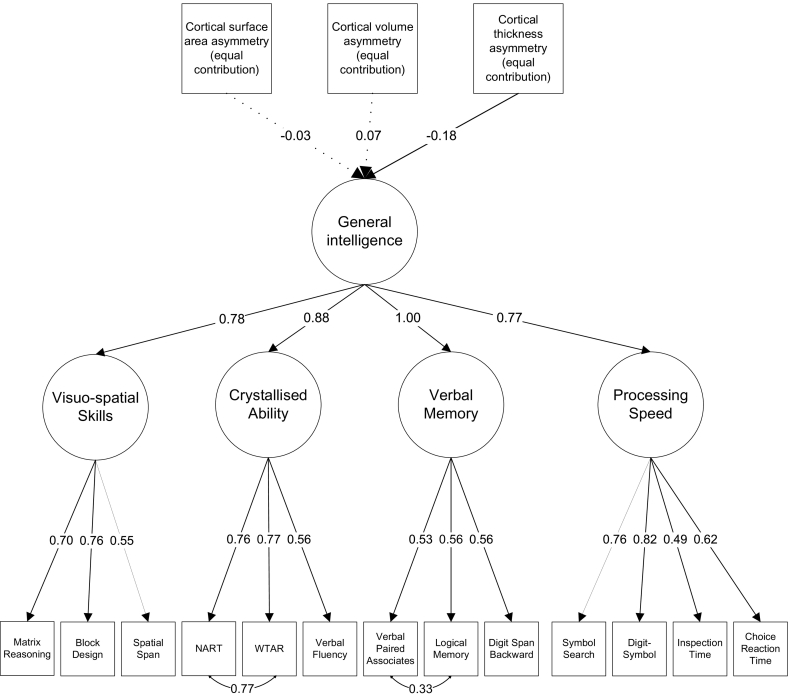
Simplified model estimating the association between cortical surface area asymmetry, volume asymmetry and thickness asymmetry (for equal-contribution asymmetry values) and general intelligence. Non-significant paths are illustrated with dotted lines.

A structural equation model was estimated to test the association between global cortical asymmetry and general intelligence (see Fig. 3). The three cortical asymmetry measures (surface area, volume and thickness) were free to correlate with each other. This model revealed a small negative association between cortical thickness asymmetry and general intelligence (*β* = −0.18, *SE* = 0.05, *p* < .001). There was no association between surface area asymmetry and general intelligence (*β* = −0.03, *SE* = 0.07, *p* = .678), or between volume asymmetry and general intelligence (*β* = 0.07, *SE* = 0.07, *p* = .286). The Bayes Factor correlations confirmed that there was strong evidence in favour of the association for thickness (BF = 1130.58; the data were over a thousand times more likely to be observed in the case of a correlation rather than the null hypothesis of no correlation). There was compelling evidence in favour of the null hypothesis for volume (BF = 0.10; the null hypothesis of no correlation was 10.27 times more likely than the alternative) and for surface area (BF = 0.11; the null hypothesis was 8.85 times more likely than the alternative).

Differences in effect sizes were analysed to investigate whether the association between thickness asymmetry and general intelligence was significantly different from the association between surface area asymmetry and/or volume asymmetry and general intelligence. As shown in Table 2, there were significant differences between the original, no constraints model (Model i) and models where equality constraints were placed on surface area asymmetry and thickness asymmetry (Model ii, *p* = .003) and volume asymmetry and thickness asymmetry (Model iii, *p* = .002). The no-constraint model had better model fit than the constrained models (e.g. AIC: Model *i* = 32,939, Model ii = 32,946, Model iii = 32,947). Therefore, the effect size of the association between thickness asymmetry and general intelligence was significantly different to the associations between surface area asymmetry and general intelligence, and volume asymmetry and general intelligence. Thus, in this sample, global thickness asymmetry was significantly more strongly related to general intelligence than was global surface area asymmetry or global volume asymmetry.

**Table 2:** Tests for differences in general intelligence effect sizes between cortical thickness asymmetry and (i) surface area asymmetry and (ii) volume asymmetry.

Model	Model constraints	χ^2^	*df*	AIC	BIC	Model of comparison	Δχ^2^	Δ *df*	Δ *p*
i	None	224.57	96	32,939	33,089	–	–	–	–
ii	Thickness asymmetry and surface area asymmetry	233.41	97	32,946	33,092	i	8.84	1	0.003
iii	Thickness asymmetry and volume asymmetry	234.00	97	32,947	33,092	i	9.43	1	0.002

#### 3.3.2 Equal regional contribution analysis: Childhood SES mediation model

**Fig. 4:**
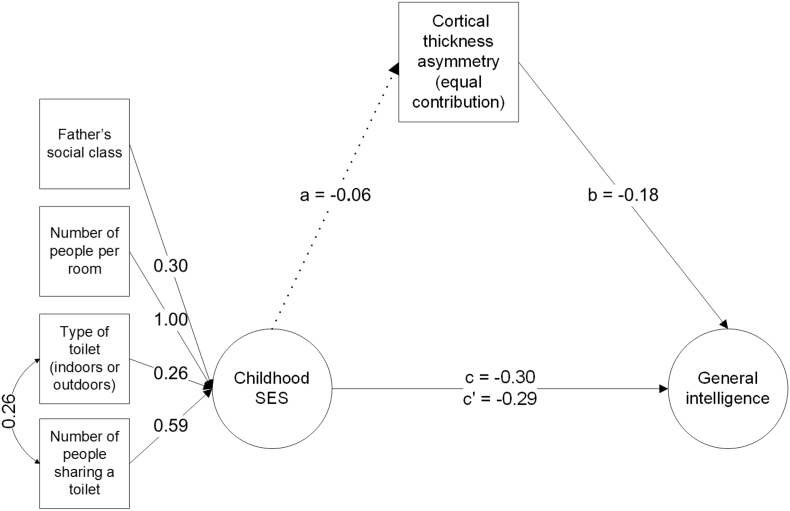
Simplified mediation model estimating the mediation of thickness asymmetry on the association between childhood SES and general intelligence. See also Fig. 3 and Supplementary Table 10.

As global cortical thickness asymmetry was significantly negatively related to general intelligence, a new model was estimated to test whether thickness asymmetry mediated the association between childhood SES and general intelligence (see Fig. 4 and Supplementary Table 10). Father's occupational class, type of toilet and number of people sharing a toilet loaded significantly (*p* < .001) on the latent factor of childhood SES. The residual variance of the path from the number of people per room to childhood SES was estimated as negative (*β* = −0.153), indicating all variance was shared with childhood SES. Therefore, as discussed above, its residual variance was set to zero. A covariance path was added between type of toilet and number of people sharing a toilet, since these variables shared significant covariance not accounted for by the paths in the rest of the model.

The bivariate association between SES and general intelligence was *β* = −0.29, *p* < .001. Whereas cortical thickness asymmetry was significantly associated with general intelligence (*β* = −0.18), it was non-significantly associated with childhood SES (*β* = −0.06, *p* = .154). The mediation model indicated that the SES-general intelligence association was not significantly mediated by cortical thickness asymmetry (attenuation 3.81%, *p* = .205, from *β* = −0.30 to *β* = −0.29).

#### 3.3.3 Equal regional contribution analysis: P-FIT versus non-Non-P-FIT asymmetry

Another aim of the present study was to investigate whether P-FIT asymmetry is more strongly related to general intelligence than non-P-FIT asymmetry, as was reported by Yeo et al. (2016). Separate values were calculated for P-FIT and non-P-FIT asymmetry. P-FIT and non-P-FIT asymmetry was moderately positively correlated on all three measures: surface area asymmetry *r* = 0.29, *p* < .001; volume asymmetry *r* = 0.24, *p* < .001; thickness asymmetry *r* = 0.29, *p* < .001. A new general intelligence model was estimated to include separate surface area asymmetry, volume asymmetry, and thickness asymmetry scores for P-FIT and non-P-FIT regions (see Table 3).

**Table 3:** *β-*values, *SE*s and *p-*values of paths from measures of cortical asymmetry to general intelligence for all regions, P-FIT and non-P-FIT regions.

	All regions	P-FIT	Non-P-FIT
Surface area asymmetry	−0.03 (0.07), *p* = .678	−0.112 (0.063), *p* = .076	0.057 (0.066), *p* = .389
Volume asymmetry	0.07 (0.07), *p* = .286)	0.038 (0.064), *p* = .549	0.047 (0.067), *p* = .483
Thickness asymmetry	−0.18 (0.05), *p* < .001	−0.068 (0.050), *p* = .173	−0.131 (0.049), *p* = .008

Importantly, we next tested formally whether P-FIT asymmetry was more strongly related to general intelligence than non-P-FIT asymmetry for cortical surface area, volume or thickness. To do this, equality constraints were placed on the P-FIT and non-P-FIT asymmetry scores for each measure in turn. For example, in Model B equality constraints were placed on P-FIT surface area asymmetry and non-P-FIT surface area asymmetry. These constrained models were compared to the original, freely-estimated, model (Model A). For each comparison, the critical *p*-value was >0.05 (see Table 4). Therefore, P-FIT asymmetry was not more strongly related to general intelligence than non-P-FIT asymmetry for cortical surface area, volume or thickness.

**Table 4:** Equality constraint comparisons between P-FIT and non-P-FIT models. Δ values refer to the difference tests between models.

Model	Model constraints	χ^2^	*df*	AIC	BIC	Model of comparison	Δχ^2^	Δ *df*	Δ *p*
A	None	261.86	132	21,793	21,956	–	–	–	–
B	P-FIT and non-P-FIT surface area asymmetry	264.70	133	21,793	21,952	A	2.84	1	0.092
C	P-FIT and non-P-FIT volume asymmetry	261.88	133	21,791	21,949	A	0.02	1	0.896
D	P-FIT and non-P-FIT thickness asymmetry	262.90	133	21,792	21,950	A	1.04	1	0.308

### 3.4 Proportional regional contribution analysis

To investigate how similar equal and proportional region contribution methods for calculating asymmetry are, correlations were conducted. The correlations were between the two overall asymmetry scores (equal and proportional) for each of the 636 participants. There was no significant correlation between equal and proportional asymmetries for surface area: *r* = −0.009 (*p* = .82). There were significant correlations for volume, *r* = 0.099 (*p* = .01), and thickness, *r* = 0.274 (*p* < .001). However, these correlations (particularly for volume) are weak. The weakness (and, in the case of surface area, non-significance) of these correlations highlights that these two methods of calculating cortical asymmetry result in very different outcomes.

**Fig. 5:**
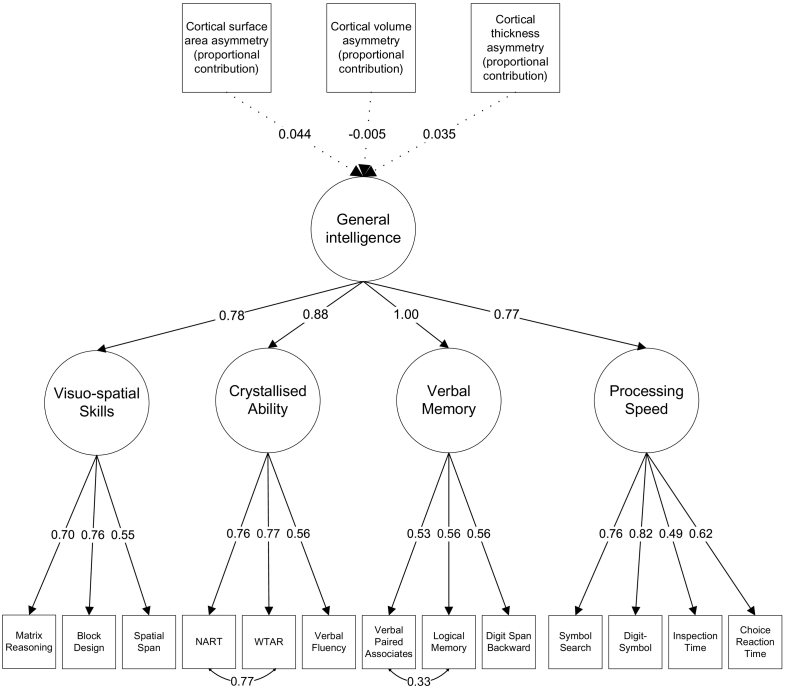
Simplified model estimating the association between cortical surface area asymmetry, volume asymmetry and thickness asymmetry (for proportional asymmetry scores) and general intelligence. Non-significant paths are illustrated with dotted lines.

Using the method of calculating asymmetry where each region contributed proportionally to the overall asymmetry score, there were no significant associations between any measures of cortical asymmetry and general intelligence (see Fig. 5). As in the equal regional contribution analysis, the three cortical asymmetry measures (surface area, volume and thickness) were allowed to correlate with each other. For all paths, *p* > .3 and *SE* = 0.046. The Bayes Factor correlations provided compelling evidence in favour of the null hypothesis for all three measures: for surface area (BF = 0.22; the null hypothesis of no correlation was 4.63 times more likely than the alternative), for volume (BF = 0.10; the null hypothesis of no correlation was 10.51 times more likely than the alternative) and for thickness (BF = 0.16; the null hypothesis was 6.34 times more likely than the alternative).

### 3.5 White matter fractional anisotropy asymmetry model

White matter fractional anisotropy asymmetry scores were calculated so that each tract contributed equally. Directional asymmetries were calculated by subtracting the right white matter tract fractional anisotropy value from the left. No absolute asymmetries were individually associated with general intelligence (all *β-*values < 0.08, all *p-*values > .05; see Supplementary Table 1). A structural equation model was estimated to test the association between global white matter fractional anisotropy asymmetry and general intelligence (see Supplementary Fig. 5). The association was small and non-significant (*β* = 0.03, *SE* = 0.05, *p* = .512). The Bayes Factor correlations confirmed that there was compelling evidence in favour of the null hypothesis (BF = 0.11; the null hypothesis of no correlation was 9.28 times more likely than the alternative). Therefore, there was no evidence that combined white matter fractional anisotropy asymmetry was significantly associated with general intelligence.

## 4 Discussion

The association between fluctuating brain asymmetry and general intelligence was estimated in a sample of older adults. Both regional and global measurements were used for grey matter (cortical volume, surface area and cortical thickness) and white matter fractional anisotropy, and general intelligence was estimated from a wide variety of tests covering several cognitive domains. The method of calculating fluctuating asymmetry made a difference to the results; it is important for future research to carefully consider and justify whether equal or proportional methods are used for calculating cortical asymmetry. There was an association between cortical thickness asymmetry and intelligence when regions contributed equally to the estimation of cortical asymmetry, but there were no such associations when the contribution of each region was proportional to its size. Cortical surface area and volume showed no significant relations to intelligence in any analysis (all *p*-values > .3). For most analyses, Bayes Factor correlations provided compelling evidence for the null hypothesis of no association between brain asymmetry and intelligence.

Using the method where regions contributed equally to the fluctuating asymmetry score, as in Yeo et al. (2016), asymmetry in global cortical thickness was significantly negatively associated with general intelligence. This association was modest (*r* = −0.18), as expected from similar asymmetry-cognitive associations (e.g. Bates, 2007; Furlow et al., 1997; Yeo et al., 2016). An exploratory analysis in a previous study suggests a positive association between cortical thickness asymmetry and working memory and vocabulary performance in young adults (*N* = 100, Plessen et al., 2014). However, like most previous studies, our finding suggested that higher fluctuating asymmetry is linked to negative cognitive outcomes (e.g. Bates, 2007; Hope, Bates, Dykiert, et al., 2013; Hope, Bates, Penke, et al., 2013). Further investigation is required, as there are age-related differences in both cortical thickness asymmetry (Thambisetty et al., 2010) and general intelligence (MacDonald, Li, & Bäckman, 2009) that might affect associations between these variables in samples of different ages. For example, Plessen et al. (2014) found that cortical thickness decreased with age in the right hemisphere but increased in the left hemisphere.

The relationship between global cortical thickness asymmetry and general intelligence can be thought of in context with relationships between fluctuating body asymmetry and cognitive performance (e.g. Bates, 2007; Furlow et al., 1997). As both brain and body asymmetries are reliably negatively associated with intelligence measures, it seems that the relationship between cortical thickness and general intelligence is unlikely to be causal. Instead, both brain and body asymmetries might be markers of developmental instability, which might impact general intelligence (van Dongen, 2006). Future work with both body and brain asymmetries could capture more variance in intelligence, and aid interpretation.

There was no association between global cortical surface area asymmetry (calculated with equal regional contributions) and general intelligence. This result appears to be inconsistent with Yeo et al. (2016; *N* = 244), who reported a significant negative association (*r* = −0.15), despite using the same methods for cortical asymmetry calculation and a full-scale IQ measure. Differences in findings could be age-related. Yeo et al. (2016) used a sample of 18–33-year-olds, whereas the current study sampled a narrower age range of approximately 73-years-old. It could be that relationships between cortical surface area measures and intelligence become harder to detect amidst the life-long accumulation of random structural influences (Dotson et al., 2016; Plessen et al., 2014). It could also be that the relationship between global cortical surface area asymmetry and intelligence changes with age. As the current study found an association between cortical thickness asymmetry and general intelligence, and Yeo et al. (2016) found an association between cortical surface area asymmetry and general intelligence, the differential genetic roots and differential ageing of cortical thickness and surface area could also explain differences in results. Whilst cortical surface area and cortical thickness are both highly heritable, they are essentially genetically unrelated (Panizzon et al., 2009) and cortical thickness is much better preserved with normal ageing, compared to surface area (Dickerson et al., 2009).

In addition, there was no significant association between cortical volume fluctuating asymmetry and general intelligence. Cortical volume is essentially the product of cortical thickness and cortical surface area and, in this sample, surface area and volume were more phenotypically similar than surface area and thickness (Cox et al., 2018). Therefore, because there was no association between surface area asymmetry and general intelligence, it follows that there was no association between volume asymmetry and general intelligence. Previous studies have not investigated a link between overall volume asymmetry and general intelligence. Instead, they have focused on specific regions and specific populations (e.g. Dougherty et al., 2016; Woolard & Heckers, 2012). Future research should investigate whether cortical volume asymmetry is associated with general intelligence in healthy young adults.

P-FIT asymmetry was not more strongly associated with general intelligence than non-P-FIT asymmetry in 73-year-olds for cortical surface area, volume or thickness. Therefore, our findings do not support Yeo et al.'s (2016) suggestion that frontoparietal surface area asymmetry predicted general intelligence whereas non-frontoparietal surface area asymmetry did not. This discrepancy could, once again, be explained by age differences: frontoparietal integrity decreases more rapidly than non-frontoparietal integrity after 60 years old (e.g. Rönnlund, Sundström, & Nilsson, 2015), and this could, in turn, affect associations between frontoparietal regions and general intelligence. Regarding the P-FIT theory, frontoparietal asymmetry does not appear to be a marker of the biological basis of general intelligence in older adults. It is possible that frontoparietal regions become less specialised for cognitive abilities in older age (see Campbell, Grady, Ng, & Hasher, 2012), making the P-FIT less meaningful in older adults. This uncertainty provides motivation for future studies to test the P-FIT separately in older adults.

Global cortical thickness asymmetry was not associated with childhood SES, providing evidence against the hypothesis that asymmetry is a significant mediator of the association between childhood SES and general intelligence. However, the participants in this study might not be representative of 73-year-olds in the general population, because they are a selective sample, who were self-motivated to participate in this research. Due to the nature of the sample selection, the effect size may have been attenuated, as there are fewer people with low SES backgrounds compared to the general population. The sample size may not have been large enough to reliably estimate the likely modest association between childhood SES and thickness asymmetry. As brain asymmetry in older age may be affected by multiple environmental factors, future research using representative samples should investigate whether childhood cortical asymmetry is associated with childhood SES.

There are differences between asymmetry for individual regions found in this study and in previous studies. For example, Wang et al. (2007) found significant directional asymmetry in the posterior cingulate in a sample of young adults. However, in the current study, there was no directional asymmetry in the posterior cingulate. Furthermore, unlike the current study, Yeo et al. (2016) found significant associations between surface area asymmetry to general intelligence in the frontal pole, caudal middle frontal, fusiform, isthmus cingulate and lingual regions. Unlike Yeo et al. (2016), the current study found significant associations from surface area asymmetry to general intelligence in the precuneus and rostral anterior cingulate regions. As the same methods were used, these findings suggest that the association between surface area asymmetry and general intelligence might change on a regional basis with age, though it could equally be that these findings are false positives or are the consequence of overfitting to the specific samples in question. To aid interpretation of these differences, future studies with longitudinal data could characterise region-based age-related changes in cortical surface area asymmetry, and also in volume and thickness. This research would be especially worthwhile in large samples covering a wide age range.

Regional and global white matter tract fractional anisotropy fluctuating asymmetries were not associated with general intelligence in 73-year-olds. It may therefore be the case that white matter fractional anisotropy asymmetry is not associated with cognitive performance. However, future research should investigate this association in younger adults. Alternatively, it could be more appropriate to investigate associations between white matter asymmetry in specific tracts and cognitive abilities relevant to them, as effects might be undetectable or negated when white matter tract asymmetry is combined. There are also other features of white matter tracts that could be investigated – for example, number of streamlines, which is a proxy measure for volume. It is also notable that our regional and global metrics were based upon a limited number of white matter pathways – these were selected due to our ability to reliably identify and measure their microstructure, but they comprise a relatively low proportion of the brain's overall white matter connective tissue. The method we used to identify tracts, probabilistic neighbourhood tractography (PNT), uses single seed point tractography to generate reliable segmentations of white matter pathways across populations. It is set up to segment 16 major tracts, including the arcuate fasciculus, all of which have clearly defined shapes which is central to the segmentation process. It would have been interesting, for example, to include the superior longitudinal fasciculus, but it cannot currently be identified using PNT, and therefore is outside the scope of the present paper.

It could be argued that weighting the cortical parcels by volume may not reflect that the ROIs can be conceived of as ‘units’ in terms of variations in cytoarchitecture and hodology. As such, developmental influence might impact each Desikan atlas ROI differently. Such a rationale may support an unweighted approach to asymmetry. However, there are many potential atlases on which to draw; whereas the Desikan atlas allows greater comparability with prior work, other atlases are variously configured to reflect cytoarchitectural regional classifications according to different brain cartographers and offer different levels of granularity (e.g. Glasser et al., 2016; Scholtens, de Reus, de Lange, Schmidt, & van den Heuvel, 2018). Current knowledge of the correspondence between gyral patterns and underlying cytoarchitecture has not converged on the Desikan atlas as necessarily optimal for demarcating the boundaries of distinct cytoarchitectural ‘units’ (e.g. Bohland, Bokil, Allen, & Mitra, 2009; Cox et al., 2014).

The current study had a large sample size (*N* = 636) compared to other studies of intelligence and brain asymmetry, and a comprehensive battery of cognitive tests. The MRI scans were completed in the same scanner at the sample clinic. The association between cortical thickness asymmetry and general intelligence was highly significant, and would survive Bonferroni correction across 166 tests. Both a strength and a limitation of the current study was the narrow age range of the sample. Whereas this enables stronger conclusions about effects in 73-year-olds, and mitigates the possibly-confounding effects of within-sample chronological age, it does not allow exploration of age-related differences, a factor which – as noted above – may moderate asymmetry-intelligence associations, yielding different results from those of previous work on asymmetry.

## 5 Conclusion

When regional measures contributed equally to fluctuating brain asymmetry scores, cortical thickness asymmetry was negatively associated (*β* = −0.18) with general intelligence in 73-year-olds. There were no associations between general intelligence and cortical surface area, cortical volume, or white matter fractional anisotropy fluctuating asymmetries. Cortical thickness asymmetry was not associated with childhood SES which did not mediate the association between childhood SES and general intelligence. There was no difference in the intelligence-cortical asymmetry association between P-FIT and non-P-FIT regions. These findings differ from Yeo et al. (2016), who found that there was a negative association between surface area asymmetry and intelligence, and that asymmetry of frontoparietal regions, but not asymmetry of non-frontoparietal regions was associated with intelligence. In contrast, when regional measures contributed proportionally to cortical hemispheric asymmetry metrics, there were no associations between cortical surface area, cortical volume or cortical thickness and general intelligence in 73-year-olds. This study raises questions about how fluctuating brain asymmetry should be measured and motivates future research to consider how best to characterise brain asymmetry.

## Appendix A Supplementary data

Supplementary material Image 1
