# Supplementary material for: Fluctuating asymmetry in brain structure and general intelligence in 73-year-olds
Source: Intelligence. 2020 Jan-Feb;78:101407. doi: 10.1016/j.intell.2019.101407 (PMC6961972; doi:10.1016/j.intell.2019.101407)
Supplement: Supplementary file 1 — Supplementary material [file mmc1.pdf]

Supplementary Material for:

Fluctuating Asymmetry in Brain Structure and General Intelligence in 73-Year-Olds

Supplementary tables

Supplementary Table 1: Descriptive statistics of separate white matter tract fractional anisotropy absolute asymmetries, and their β-weights to general intelligence. All β-weights were non-significant ($p > .05$).

White matter tract	*N* (Left)	*N* (Right)	*M* (*SD*)	β-weight to general intelligence
Arcuate	639	580	.01 (.01)	-.04
Anterior thalamic radiations	556	643	.01 (.003)	.02
Bilateral cingulum cingulate gyri	641	650	.11 (.09)	.001
Uncinate	567	628	.14 (.11)	-.05
Inferior longitudinal fasciculi	628	664	.11 (.09)	.08

Supplementary Table 2: Results from models that excluded MRI abnormalities.

	Surface area	Volume	Thickness
Equal contributions	β = 0.008, *SE* = 0.07, *p* = .914	β = 0.12, *SE* = 0.08, *p* = .125	β = -0.19, *SE* = 0.05, *p* < .001
Proportional contributions	β = 0.08, *SE* = 0.05, *p* = .115	β = 0.003, *SE* = 0.05, *p* = .945	β = -0.007, *SE* = 0.05, *p* = .893

Supplementary Table 3: Means and *SD*s for left and right hemispheres for cortical surface area, volume and thickness.

Cortical region	Surface area (mm^2)		Volume (mm^3)		Thickness (mm)	
	Left *M* (*SD*)	Right *M* (*SD*)	Left *M* (*SD*)	Right *M* (*SD*)	Left *M* (*SD*)	Right *M* (*SD*)
Banks of the superior temporal sulcus	887.07 (176.45)	815.68 (123.70)	1967.44 (433.66)	1922.69 (364.69)	2.13 (0.21)	2.27 (0.21)
Caudal anterior cingulate	573.26 (111.15)	670.79 (132.19)	1421.54 (414.88)	1674.57 (455.69)	2.34 (0.40)	2.25 (0.34)
Caudal middle frontal	2057.80 (321.87)	1931.41 (315.62)	5300.53 (898.39)	5010.71 (905.37)	2.29 (0.17)	2.28 (0.17)
Cuneus	1359.04 (201.45)	1413.75 (199.53)	2576.17 (443.63)	2703.91 (454.17)	1.74 (0.14)	1.75 (0.15)
Entorhinal	384.15 (74.77)	338.75 (79.03)	1772.26 (416.12)	1672.95 (401.35)	2.99 (0.48)	3.16 (0.50)
Frontal pole	219.70 (36.98)	294.80 (46.55)	788.58 (163.89)	1024.96 (206.90)	2.63 (0.32)	2.55 (0.29)

Fusiform	2915.61	2818.91	8493.70	8154.62	2.38	2.38
	(407.09)	(382.98)	(1382.68)	(1277.06)	(0.19)	(0.19)
Inferior parietal	4096.06	4809.82	11002.15	13002.18	2.30	2.33
	(407.09)	(629.75)	(1382.68)	(1737.65)	(0.16)	(0.16)
Inferior temporal	2936.59	2777.45	9652.79	9322.60	2.61	2.64
	(407.09)	(405.39)	(1522.56)	(1444.17)	(0.20)	(0.21)
Insula	2030.83	2039.06	6518.68	6544.72	2.92	2.93
	(222.96)	(274.90)	(769.15)	(1444.17)	(0.19)	(0.19)
Isthmus cingulate	952.04	876.92	2410.87	2215.81	2.40	2.37
	(154.60)	(136.97)	(370.86)	(356.94)	(0.22)	(0.23)
Lateral occipital	4477.62	4321.30	10484.21	10624.96	2.08	2.17
	(543.53)	(523.59)	(1463.77)	(1475.80)	(0.15)	(0.16)
Lateral orbitofrontal	2237.17	2224.81	6542.66	6418.76	2.50	2.49
	(310.14)	(301.89)	(767.42)	(758.56)	(0.19)	(0.19)
Lingual	2729.36	2780.27	5675.67	5809.54	1.85	1.87
	(368.50)	(363.12)	(825.43)	(852.18)	(0.13)	(0.13)
Medial orbitofrontal	1685.51	1618.71	4910.28	4652.62	2.52	2.40
	(246.22)	(233.80)	(692.77)	(618.61)	(0.21)	(0.23)
Middle temporal	2746.85	3027.31	8929.82	10148.88	2.55	2.63
	(387.81)	(392.31)	(1368.09)	(1440.48)	(0.19)	(0.18)
Parahippocampal	641.80	613.79	1733.77	1589.82	2.16	2.10
	(107.99)	(93.26)	(375.98)	(312.77)	(0.37)	(0.32)
Paracentral	1229.38	1394.31	3030.94	3348.18	2.18	2.15
	(165.29)	(185.61)	(486.50)	(534.99)	(0.17)	(0.17)
Pars opercularis	1477.65	1254.60	3965.81	3337.00	2.29	2.31
	(221.72)	(194.24)	(661.38)	(564.48)	(0.15)	(0.15)
Pars orbitalis	579.37	701.70	1866.44	2248.28	2.44	2.46
	(77.76)	(99.09)	(275.13)	(341.98)	(0.22)	(0.22)
Pars triangularis	1146.92	1325.72	2909.65	3444.06	2.16	2.20
	(164.90)	(213.49)	(468.49)	(602.54)	(0.16)	(0.16)
Pericalcarine	1226.20	1357.73	1772.91	1972.25	1.50	1.49
	(215.37)	(231.18)	(351.02)	(379.21)	(0.14)	(0.14)
Postcentral	3982.52	3847.40	8427.31	7972.79	1.85	1.83
	(437.97)	(459.67)	(1062.01)	(1110.17)	(0.12)	(0.13)
Posterior cingulate	1023.76	1026.73	2666.34	2661.67	2.38	2.35
	(134.74)	(148.89)	(437.03)	(426.33)	(0.22)	(0.21)
Precentral	4530.40	4549.54	11252.33	11168.51	2.25	2.23
	(478.86)	(490.06)	(1301.43)	(1325.42)	(0.17)	(0.17)
Precuneus	3367.85	3520.62	8219.03	8415.09	2.15	2.14
	(399.04)	(443.47)	(1042.23)	(1073.91)	(0.15)	(0.14)
Rostral anterior	697.66	563.16	2274.40	1819.22	2.79	2.76
cingulate	(157.11)	(130.26)	(495.03)	(453.10)	(0.30)	(0.34)
Rostral middle	4891.62	5150.11	12467.16	13065.35	2.15	2.14
frontal	(672.51)	(756.24)	(1633.68)	(1789.28)	(0.14)	(0.14)
Superior frontal	6460.72	6269.43	18961.67	18241.14	2.45	2.42
	(735.87)	(739.08)	(2122.46)	(2112.27)	(0.15)	(0.14)
Superior parietal	5031.29	5051.21	11619.40	11612.34	2.01	2.00
	(574.64)	(585.32)	(1620.43)	(1669.89)	(0.18)	(0.16)
Superior temporal	3482.51	3319.09	9564.62	9420.31	2.30	2.36
	(401.16)	(356.92)	(1331.14)	(1316.02)	(0.18)	0.19)
Supramarginal	3542.61	3398.46	9331.00	8931.64	2.29	2.31
	(485.35)	(481.25)	(1251.36)	(1280.49)	(0.15)	(0.16)
Temporal pole	499.79	446.34	2515.83	2315.12	3.36	3.48
	(61.05)	(63.81)	(442.25)	(433.92)	(0.35)	(0.37)

Transverse temporal	423.04 (69.75)	316.11 (52.58)	1000.71 (179.54)	779.84 (158.22)	2.12 (0.22)	2.19 (0.24)

Supplementary Table 4: Descriptive statistics for absolute cortical asymmetries (surface area, volume and thickness) for specific brain regions.

	Surface area asymmetry *M* (*SD*)	Volume asymmetry *M* (*SD*)	Thickness asymmetry *M* (*SD*)
Bank superior temporal sulcus	.07 (.07)	.09 (.07)	.04 (.03)
Caudal anterior cingulate	.09 (.07)	.15 (.11)	.08 (.06)
Caudal middle frontal	.06 (.05)	.06 (.05)	.02 (.02)
Cuneus	.05 (.04)	.06 (.05)	.03 (.03)
Entorhinal	.08 (.08)	.10 (.08)	.06 (.05)
Frontal pole	.07 (.06)	.09 (.07)	.05 (.04)
Fusiform	.04 (.03)	.05 (.04)	.03 (.02)
Inferior parietal	.04 (.03)	.04 (.04)	.02 (.02)
Inferior temporal	.04 (.03)	.05 (.04)	.03 (.02)
Insula	.05 (.03)	.04 (.03)	.02 (.02)
Isthmus cingulate	.05 (.04)	.06 (.05)	.04 (.03)
Lateral occipital	.04 (.03)	.04 (.04)	.02 (.02)
Lateral orbitofrontal	.03 (.02)	.03 (.03)	.03 (.02)
Lingual	.04 (.03)	.05 (.04)	.03 (.02)
Medial orbitofrontal	.05 (.04)	.05 (.04)	.03 (.03)
Middle temporal	.04 (.03)	.05 (.04)	.02 (.02)
Parahippocampal	.06 (.06)	.09 (.07)	.06 (.05)
Paracentral	.05 (.04)	.06 (.05)	.03 (.02)
Pars opercularis	.06 (.05)	.09 (.07)	.06 (.05)
Pars orbitalis	.05 (.04)	.06 (.05)	.02 (.02)
Pars triangularis	.06 (.05)	.06 (.05)	.04 (.03)
Pericalcarine	.05 (.04)	.07 (.05)	.03 (.02)
Postcentral	.03 (.03)	.06 (.05)	.03 (.03)
Posterior cingulate	.05 (.04)	.04 (.04)	.02 (.02)
Precentral	.03 (.02)	.06 (.05)	.04 (.03)
Precuneus	.03 (.02)	.03 (.03)	.02 (.02)
Rostral anterior cingulate	.09 (.07)	.03 (.02)	.02 (.02)
Rostral middle frontal	.05 (.03)	.10 (.08)	.06 (.05)
Superior frontal	.03 (.02)	.05 (.03)	.02 (.02)
Superior parietal	.03 (.03)	.03 (.02)	.02 (.01)
Superior temporal	.03 (.02)	.04 (.03)	.02 (.02)
Supramarginal	.05 (.04)	.04 (.03)	.02 (.02)
Temporal pole	.06 (.05)	.05 (.04)	.02 (.02)
Transverse temporal	.06 (.05)	.07 (.06)	.04 (.03)

Supplementary Table 5: Absolute fit indices for the four theoretical models estimated in the current study.

Model	χ^2	*df*	CFI	TLI	RMSEA	SRMR
General intelligence model	267.63	135	0.96	0.95	0.06	0.05
Equal-contribution cortical asymmetry and general intelligence model	224.57	96	0.96	0.95	0.05	0.05
Mediation model (including childhood SES)	294.69	129	0.97	0.95	0.05	0.06
Proportional contribution cortical asymmetry and general intelligence model	213.98	96	0.96	0.96	0.05	0.04
White matter fractional anisotropy asymmetry and general intelligence model	191.82	192	0.96	0.95	0.05	0.05

Supplementary Table 6: Male-female measurement invariance tests (configural, weak and strong) for the general intelligence model.

Model number	Model description	χ^2	*df*	AIC	BIC	Model of comparison	**Δ** χ^2	**Δ** *df*	**Δ** *p*
1	Configural invariance	237.33	120	45390	45777	-	-	-	-
2	Weak invariance (equal loadings)	258.29	132	45387	45721	1	20.96	12	.051
3	Strong invariance (equal loadings and equal intercepts)	313.14	140	45425	45725	1	54.86	8	<.001

Supplementary Table 7: Sex differences in cognitive tests.

Cognitive domain	Test	*N*	Overall *M* (*SD*)	M_{male} (*SD*)	M_{female} (*SD*)	Sex difference *p*	Sex difference *d*
Visuospatial Skills	Matrix Reasoning	634	13.52 (4.93)	14.00 (4.92)	12.98 (4.90)	.009	.21
	Block Design	634	34.38 (10.01)	35.56 (10.71)	33.07 (9.00)	.002	.25
	Spatial Span	634	14.79 (2.72)	15.09 (2.83)	14.45 (2.56)	.003	.24
Crystallised Ability	NART	634	34.66 (8.10)	33.67 (8.52)	35.77 (7.46)	.001	-.26

	WTAR	634	41.27 (6.94)	40.59 (7.70)	42.03 (5.89)	.008	-.21
	Phonemic Verbal Fluency	635	43.55 (12.78)	42.55 (13.43)	44.68 (11.93)	.035	-.17
Verbal Memory	Verbal Paired Associates	623	27.57 (9.48)	25.95 (9.77)	29.36 (8.84)	<.001	-.36
	Logical Memory	635	75.03 (17.84)	73.49 (18.59)	76.76 (16.83)	.020	-.18
	Digit span backwards	636	7.88 (2.31)	7.74 (2.36)	8.04 (2.23)	.095	-.13
Processing Speed	Symbol Search	634	24.88 (6.05)	24.59 (6.25)	25.20 (5.82)	.204	-.10
	Digit-Symbol Substitution	634	56.68 (11.79)	54.53 (11.88)	59.09 (11.23)	< .001	-.39
	Inspection Time	624	111.78 (10.95)	112.73 (11.38)	110.70 (10.34)	.020	.19
	Four-Choice Reaction Time	635	0.64 (0.08)	0.65 (0.09)	0.64 (0.08)	.280	-.09

Participants performed above WAIS-III and WMS-III manual norms for 70-74 year olds on all tests, apart from the Symbol Search test, where they performed at average (Wechsler, 1997a, 1997b). This indicates that they are not a representative sample of the general population. *t*-tests revealed significant sex differences in 10 of the 13 cognitive tests (see Table 2). Generally, the sex differences found here are consistent with those reported in previous research (e.g. Delgado & Prieto, 1996; Krueger & Salthouse, 2010; Lowe, Mayfield & Reynolds, 2003). Males tended to perform better at visuospatial tasks and females tended to perform better at vocabulary-based tasks.

Supplementary Table 8: Path *β*-weight values, *SE*s, *p*-values and residual variances for the general intelligence model. *** < .001.

Cognitive domain	Domain *β* (*SE*)	*p*	Domain residual variances	Test	Test β (*SE*)	*p*	Test residual variance
Visuo-spatial	.78 (.03)	***	0.39	Matrix Reasoning	.70 (.03)	***	.50
				Block Design	.76 (.03)	***	.43
				Spatial Span	.55 (.04)	***	.70
Crystallised	.88 (.04)	***	0.23	NART	.76 (.03)	***	.42
				WTAR	.77 (.03)	***	.40
				Phonemic Verbal Fluency	.56 (.04)	***	.69

Verbal memory	1.00 (.00)	-	0.00	Verbal Paired Associates	.53 (.04)	***	.72
				Logical Memory	.56 (.03)	***	.69
				Digit span backward	.56 (.03)	***	.69
Speed	.77 (.03)	***	0.41	Symbol Search	.76 (.02)	***	.42
				Digit-Symbol Substitution	.82 (.02)	***	.32
				Inspection Time	.49 (.04)	***	.76
				Four-Choice Reaction Time	.62 (.03)	***	.62

Supplementary Table 9: Path *β*-weight values for individual cortical regions and general intelligence. Unless otherwise specified, $p > .05$. Significant *β*-weights are in boldface text.

	Surface area Asymmetry	Volume asymmetry	Thickness asymmetry
Banks of the superior temporal sulcus	.01	.07	.002
Caudal anterior cingulate	.07	.02	-.07
Caudal middle frontal	-.02	.04	-.007
Cuneus	-.04	-.06	-.02
Entorhinal	.02	.01	-.04
Frontal pole	-.07	-.07	-.04
Fusiform	-.09	-.05	.02
Inferior parietal	-.02	.02	.02
Inferior temporal	.06	**.10** **$p = .034$**	-.005
Insula	-.05	-.04	-.01
Isthmus cingulate	-.02	-.02	-.06
Lateral occipital	-.002	.06	-.04
Lateral orbitofrontal	-.06	-.07	-.03
Lingual	-.05	-.06	-.02
Medial orbitofrontal	.02	.001	-.04
Middle temporal	.02	.05	.03
Parahippocampal	.04	.02	-.02
Paracentral	.06	.06	-.009
Pars opercularis	.01	.02	-.04
Pars orbitalis	-.07	-.02	-.05
Pars triangularis	.07	.08	.04
Pericalcarine	-.08	-.03	-.09
Postcentral	-.07	-.01	-.007
Posterior cingulate	-.08	-.09	.02

Precentral	-.07	-.07	.02
Precuneus	**.13** **_p_ = .007**	-.01	-.06
Rostral anterior cingulate	**.13** **_p_ = .004**	.08	-.03
Rostral middle frontal	.04	-.01	-.04
Superior frontal	.08	.09	.005
Superior parietal	-.02	-.03	-.03
Superior temporal	.002	-.07	-.07
Supramarginal	-.02	-.04	.03
Temporal pole	-.02	-.06	-.02
Transverse temporal	**.09** **_p_ = .047**	.09	.04

Supplementary Table 10: Descriptive statistics, path *β*-weight values, *SE*s, *p*-values and residual variances for the childhood SES latent factor in the exploratory mediation model.

	N	*M* (*SD*)	β (*SE*)	*p*	Residual variance
Father's social class	584	2.92 (0.91)	.30 (.04)	<.001	.90
Number of people per room	630	1.34 (0.74)	1.00 (.00)	-	.00
Toilet type	632	1.11 (0.32)	.26 (.04)	<.001	.92
Number of people sharing a toilet	627	5.28 (2.54)	.59 (.03)	<.001	.62

Supplementary figures

Supplementary Figure 1: Summary of directional asymmetries for cortical surface area, volume and thickness of cortical regions.

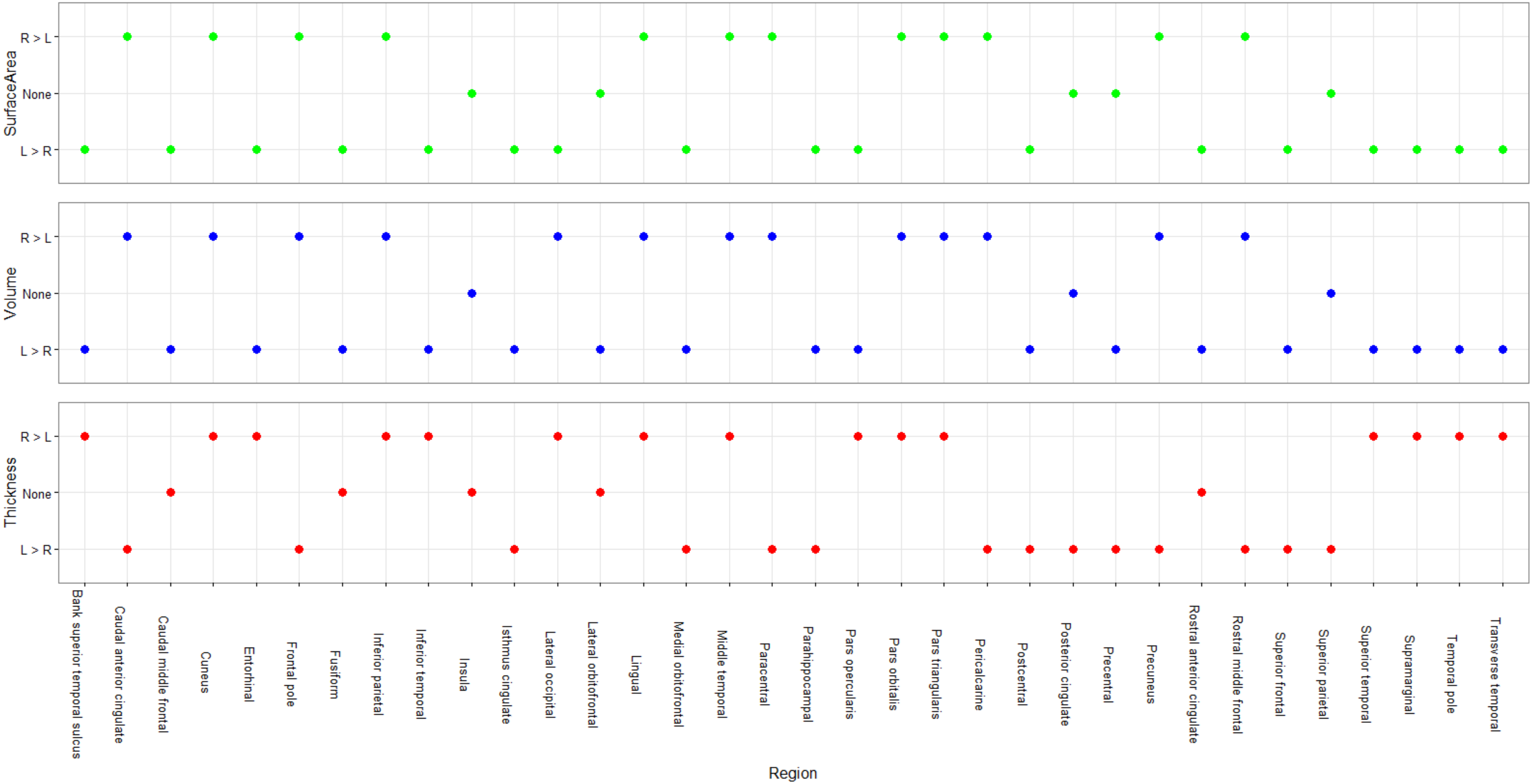

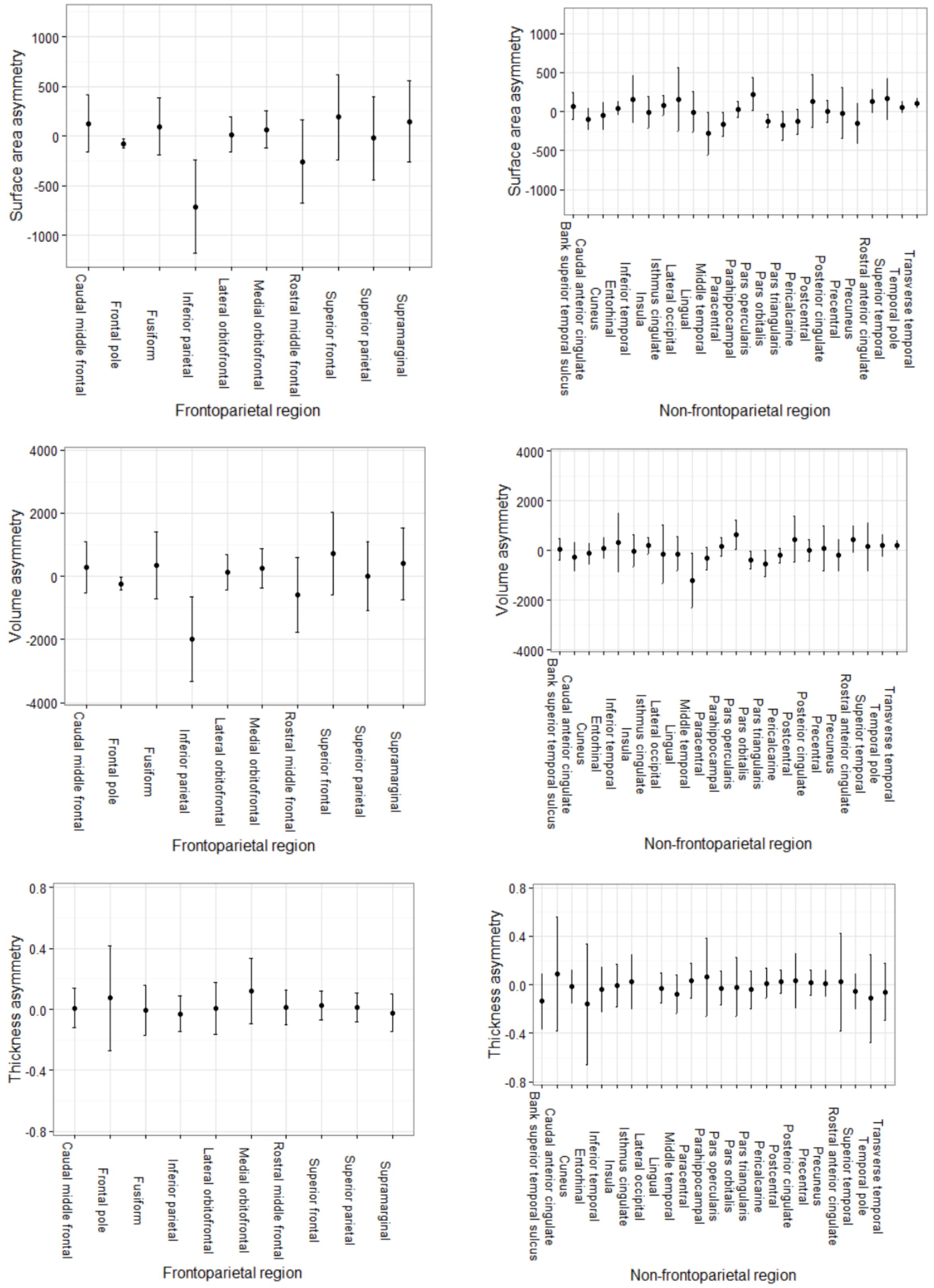

Supplementary Figure 2: P-FIT and non-P-FIT mean directional asymmetry graphs by region. This figure shows each region's mean directional asymmetry and *SE* for P-FIT and non-P-FIT regions.

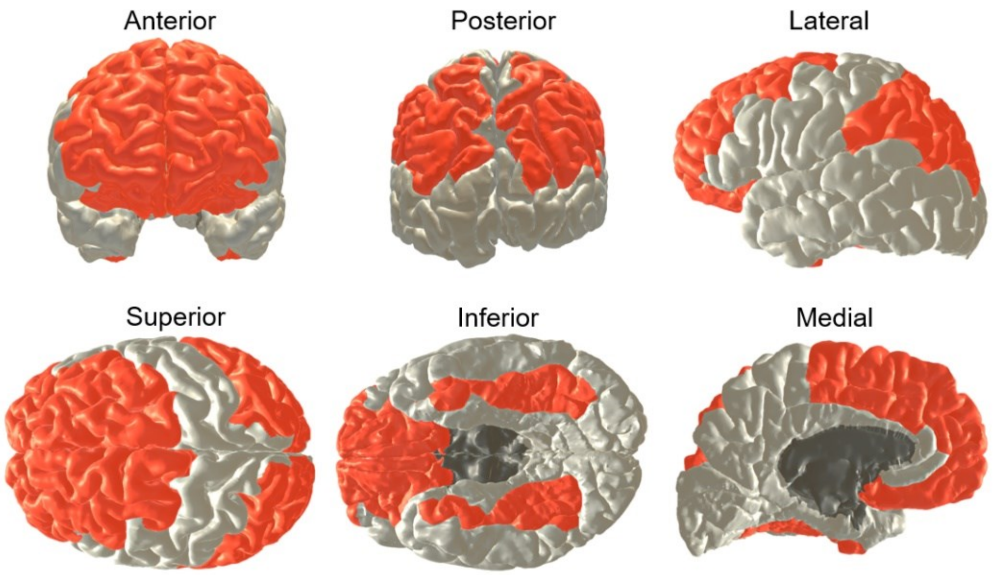

Supplementary Figure 3: Illustration of P-FIT (red) and non-P-FIT (grey) regions.

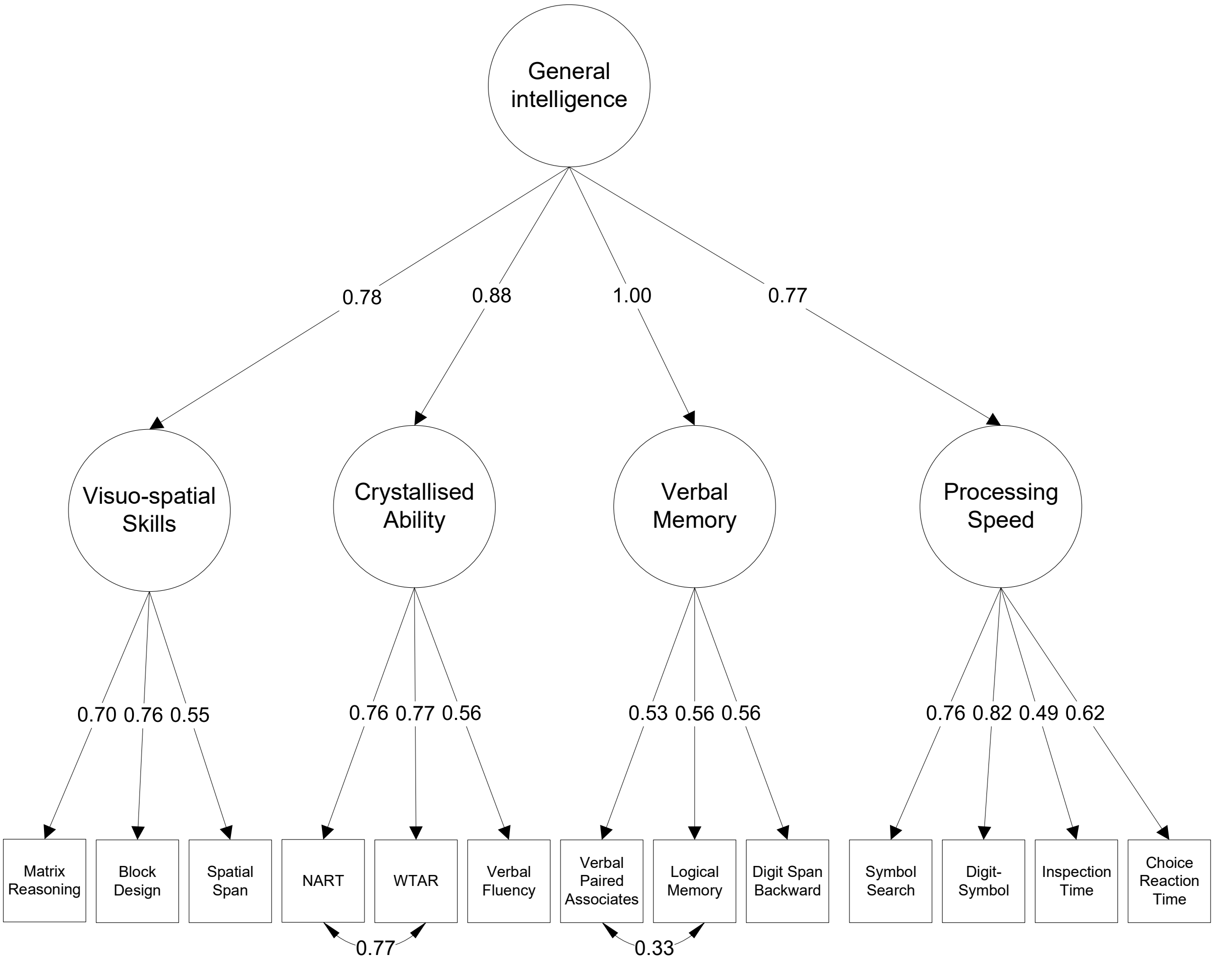

Supplementary Figure 4: Simplified model of general intelligence. For full details, including *SE*s, residual variances and *p*-values, see Appendix 1.

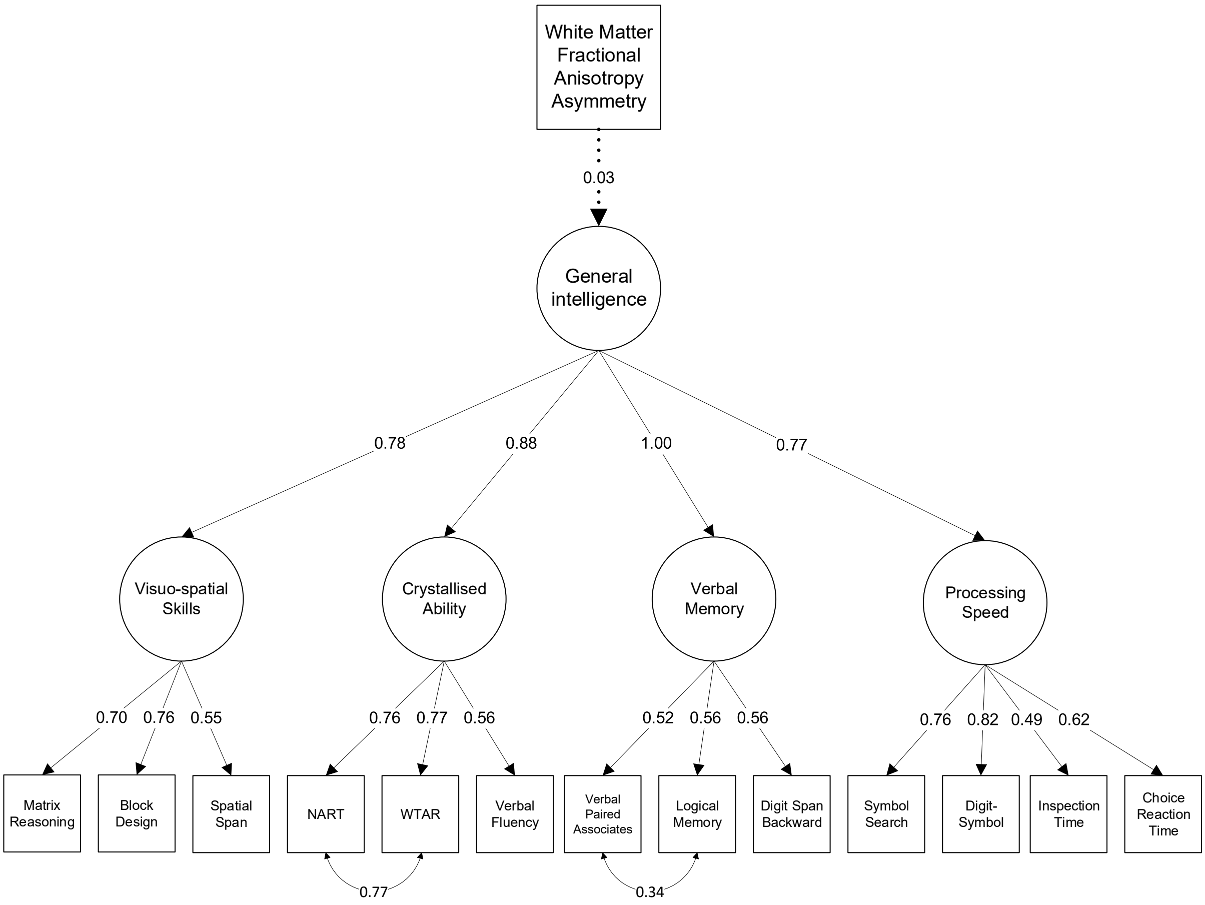

Supplementary Figure 5. Simplified diagram of model estimating the association between white matter fractional anisotropy asymmetry and general intelligence. Non-significant paths are illustrated with dotted lines. Residual variances for each variable are not shown.

<u>References</u>

Delgado, A. R., & Prieto, G. (1996). Sex-difference in visuospatial ability: Do performance factors play such an important role? *Memory and Cognition*, 24(4), 504-510.

Krueger, L. E., & Salthouse, T. A. (2010). Differences in acquisition, not retention, largely contribute to sex-differences in multi-trial word recall performance. *Personality and Individual Differences*, 49, 769-772.

Lowe, P. A., Mayfield, J. W., & Reynolds, C. R. (2003). Gender differences in memory test performance among children and adolescents. *Archives of Clinical Neuropsychology*, 18(8), 865-878.
